# Supplementary material for: Are carbon emissions trading and green financial instruments synergistic? -Comprehensive quantitative research based on content analysis
Source: PLoS One. 2024 Mar 7;19(3):e0298601. doi: 10.1371/journal.pone.0298601 (PMC10919601; doi:10.1371/journal.pone.0298601)
Supplement: S1 Data — (ZIP) [file pone.0298601.s001.zip › Data/地级市Z_F工作报告.zip!/╔┬╬≈/▒a╝a╩╨/└┤╫╘╣1⁄2╓┌║┼í╛┬φ┐╦╩2╛▌═°í┐_2012.docx]

各位代表：

现在，我代表市人民政府向大会作工作报告，请予审议，并请市政协委员和其他列席人员提出意见。

过去四年工作回顾

过去的四年，在市委的正确领导下，我们深入贯彻落实科学发展观，抢抓新一轮西部大开发和关天规划实施机遇，坚持“四个第一”，推进“五大突破”，强力实施“率先发展八大工程”，奋力抓好“十件大事”，全面完成了各项预定目标任务。

经济保持快速增长，综合实力稳步提升。四年来，地区生产总值年均增长14.8%，2011年全市实现地区生产总值1175.75亿元，首次突破千亿元大关；财税总收入达到151.7亿元，其中地方财政收入51.4亿元；城镇居民人均可支配收入、农村居民人均纯收入分别达到22337元、6340元。12户大企业大集团总产值占到规模以上工业的53.3%，东岭集团、宝石钢管、宝钛集团实现产值过百亿元；汽车及零部件、钛及钛合金、石油装备三个产业集群初具规模，机床工具、酒类等六个产业集群加速崛起。2011年，全市规模以上工业实现增加值534.3亿元，增长19.3%。高新区实现地区生产总值375亿元，综合排名位列国家级高新区第37位。周公庙、中华礼乐城、凤凰湖先后晋升为国家4A级景区，成功举办“两节一会”、西交会和全球华人省亲祭祖大会等重大节庆活动，旅游综合收入四年累计达到376亿元。

项目建设深入实施，发展基础更加稳固。重大产业项目取得突破，陕汽30万辆微型车一期、大唐热电、千阳海螺水泥、凤翔冀东水泥等项目建成投产，宝钛工业园、宝石钢管工业园、秦川铸造工业园、国核锆材、航空装备产业园等项目进展顺利。重点交通项目强力推进，西宝高速改扩建工程顺利竣工，宝天高速、宝平高速宝鸡段建成通车，西宝客运专线、麟北煤田铁路专线开工建设。商贸旅游项目亮点纷呈，法门寺文化景区一期、中华礼乐城、中华石鼓园和宝鸡会展中心、开元商城、大润发超市、新冠森国际家居建材生活馆等项目相继建成运营。招商引资成效明显，成功引进了深圳东部集团、海航集团、南车时代、西电集团、华泽集团等一大批国内知名企业。四年累计引进5亿元以上项目96个，实现到位资金1321亿元；完成全社会固定资产投资2933亿元，年均增长36.6%。

城乡建设成效显著，人居环境日益优越。编制完成了新一轮城市总体规划和土地利用规划，建成区基础设施不断完善提升，高新区三期建设加快推进，代家湾生态大道、蟠龙塬新区开发正式启动，发展空间进一步拓展。蟠龙、蔡阳、龚刘、凤凰等渭河大桥建成通车，高速客运站、新滨河南路、宝钛路、宝福路、川陕路、陈仓大道改造等项目顺利完成。老城区集中供热改造、5万吨中水回用、渭河南截污工程等项目竣工投用，各县污水处理厂、生活垃圾处理场全部建成，天然气利用实现市域全覆盖。在西北地区首家开通了数字化城管系统，被列为全国数字化城市管理试点城市。先后荣获全国节水型城市、国家森林城市、中国人居环境奖、全国环境优美城市等多项荣誉，国家生态示范市通过考核验收，国家园林城市、国家卫生城市通过复检，生态建设走在西部前列。成功创建国家卫生县城8个、国家级生态示范县区10个、全国绿化模范县3个、国家园林县城4个，省级园林县城实现全覆盖。

统筹城乡步伐加快，农村面貌焕然一新。粮食生产实现“八连增”，2011年总产达到142.5万吨。奶牛存栏18.8万头，稳居全省第一，现代牧业两万头奶牛场、13个千头奶牛场建成投用。果菜产业突破发展，水果、蔬菜面积分别达到89.3万亩和74万亩，核桃113万亩，花椒86万亩。农业科技支撑能力进一步增强，12个农业科技示范园区建设扎实推进，建成国家甘薯、肉牛、肉羊综合试验站。农业产业化水平持续提升，市级以上龙头企业达到184户，其中亿元以上企业21户，发展农民专业合作社1113个。自来水普及率位居全省首位，解决了95万农村人口的饮水安全问题，渭河整治、病险水库除险加固、中小河流治理取得阶段性成果，通村公路实现全覆盖。新农村“晋星创建”活动扎实开展，全市四星村、五星村分别达到437个和163个，农村生产生活条件全面改善。突破西山板块扶贫模式在全省推广，全市35万农村贫困人口实现脱贫，荣获全国扶贫开发先进集体。灾后重建任务全面完成，累计投资58.5亿元。统筹城乡深入推进，凤县省级试点，高新区、蔡家坡市级试点初见成效，全市16.2万农村居民进城落户。凤县进入西部百强县，凤翔县跨入全省十强县，渭滨区跻身全省五强区，东岭村位列“中国经济十强村”第4名。

改革开放深入推进，发展活力不断增强。国有企业改革迈出新步伐，烽火集团实现借壳上市，宝鸡机床与秦川集团，宝光集团、陕开集团与西电集团实现强强联合，西凤酒、红旗民爆等企业完成增资扩股，33户企业完成政策性破产、依法破产和改制重组。成功引进交通、中信、民生银行和齐鲁证券等金融机构，在全省首家成立硕丰村镇银行，陇县合作银行成为关天经济区第一家农村合作银行，2011年全市实际利用信贷资金达到604.5亿元，较四年前翻了一番，荣获中国金融生态城市称号。集体林权主体改革、市县政府机构改革和撤乡并镇工作全面完成，高新区、蔡家坡经开区管理体制改革取得突破。对外贸易大幅增长，外贸总量稳居全省第二，出口增速名列全省前茅。

社会事业全面发展，民生保障持续改善。无缝隙全覆盖的社会保障制度基本建立，初步形成了社会保障宝鸡模式，在全国率先启动了新型农村养老保险、城镇居民养老保险，并实现了城乡并轨。城乡居民养老、城镇职工医疗、城镇居民医疗、新型农村合作医疗保险参保率分别达到95%、97%、95%和98%。城乡低保、大病救助等社会救助体系日益完善，救助水平不断提高。创业就业工作成效显著，建成各类创业基地46个，被确定为全国统筹城乡就业试点城市和创业型示范城市，每年转移农村劳动力100万人以上，四年累计新增城镇就业21.9万人，城镇登记失业率控制在4%以内。累计开工建设保障性住房8.35万套652万平方米，连续四年被评为全省住房保障工作先进城市。教育投入持续增长，办学条件大幅改善，教育质量稳步提高，“双高双普”和“教育强县”创建工作走在全省前列，宝鸡文理学院荣获硕士学位授权单位立项建设高校，宝鸡职业技术学院入围全省示范性高职院校创建行列。创新能力得到加强，被确定为国家创新型试点城市，跻身国家知识产权示范市、全国科技进步考核先进市，石油天然气管材、油气钻井装备两个国家级工程技术研究中心获批组建。医药卫生体制改革成效显著，公立医院重大体制机制改革实现新突破，医疗卫生服务体系更加完善，基本药物制度和零差率销售实现基层医疗机构全覆盖，食品药品监测整治工作进入全国先进行列，城乡居民主要健康指标高于全省平均水平。创建国家计生优质服务先进县区7个，实现省级计生优质服务先进县全覆盖，被确定为全国人口计生综合改革示范市。文化体制改革任务全面完成，图书馆、文化馆、博物馆免费向社会开放，成为首批国家公共文化服务体系示范区创建城市。全国文明城市创建工作扎实推进，凤县、凤翔成为全国文明县城。竞技体育与群众体育水平全面提升，成功举办了全国武术散打冠军赛、环中国国际公路自行车赛等15项国际、国家级赛事，取得省第十五届运动会承办权，成为全国全民健身示范试点城市。全国双拥模范城“七连冠”通过国家检查验收,荣获全国人民防空先进城市、全国残疾人工作示范城市称号，老龄、审计、地方志、关心下一代工作受到国家部委表彰。人事、监察、国土、统计、档案、物价、地震、气象、慈善以及民族宗教、国家安全、外事侨务和妇女儿童等工作都取得了新成绩。

四年来，我们不断加强民主法制建设和政府自身建设，自觉接受市人大、市政协的监督，广泛听取人大代表、政协委员、民主党派以及社会各界的意见、建议。“五五”普法高标准通过验收，“六五”普法全面启动。应急管理体制机制不断完善，处置突发公共事件能力进一步增强。社会治安综合治理继续深化，“平安宝鸡”创建成效明显，一批信访突出问题得到解决，安全生产形势总体平稳，社会治安满意率保持在90%以上，位居全省前列，社会大局和谐稳定。

各位代表，过去的四年，是宝鸡发展历史上极不平凡的四年。我们成功应对冰雪地震自然灾害，努力克服金融危机不利影响，共同谱写了宝鸡发展历程上的光辉篇章。四年来，经过全市上下的共同努力，综合实力跃上新的台阶，地区生产总值实现了翻番，跻身新中国成立60周年60个城市发展代表行列；工业经济进一步壮大，规模以上工业总产值实现了翻番，突破千亿元大关；大城市建设实现新的跨越，关中西部城镇群建设加快推进，城镇化率达到44.7%；社会保障全面覆盖，社会事业蓬勃发展，人民幸福指数明显提升；生态环境建设成效显著，社会管理不断创新，城乡社会更加和谐。这些成绩的取得，是市委总揽全局、正确领导的结果，是市人大、市政协鼎力支持、共谋发展的结果，是全市人民同心同德、顽强拼搏的结果。在此，我代表市政府，向给予我们支持和监督的人大代表、政协委员、各民主党派、工商联、各人民团体、社会各界人士，向驻宝企事业单位、解放军和武警部队，表示崇高的敬意和衷心的感谢！

在肯定成绩的同时，我们也要清醒地看到，在发展中还面临着一些突出矛盾和问题，主要是：经济总量不大，2011年仅占全省的9.5%，距离省上要求12%的目标还有较大差距；工业实力不强，九大产业集群无一突破500亿元；对项目建设和招商引资重视不够，解决项目少、项目小的办法不多；第三产业发展滞后，非公经济发展较慢，市强县弱的经济格局仍未改变，三产增加值、非公经济总量、县域经济总量分别占全市生产总值的25.4%、49.2%、46.4%；城镇化水平不高，城镇化率分别低于全国、全省6.6个百分点和2.3个百分点；少数政府工作人员服务意识不强，工作作风不实，群众满意度较低，与新的发展形势还不相适应。对于这些问题，我们将高度重视，采取有力措施，切实加以解决，绝不辜负全市人民的期望和重托，努力开创宝鸡发展的新局面！

今后五年的奋斗目标和重点任务

未来五年，是我市全面实现“十二五”规划各项目标任务、打造关天副中心城市的关键时期，也是进一步发挥优势、加快建设小康社会的重要阶段。当前，国际经济形势依然严峻，世界经济复苏乏力；国内经济增长下行压力较大、增速放缓，潜在风险增多。但是，我们也应看到，国家将突出稳中求进的总基调，保持经济政策的连续性和稳定性，继续实施积极的财政政策和稳健的货币政策，经济发展环境总体向好。纵观宝鸡发展，经过改革开放30多年的建设和积累，综合实力显著增强，加快发展的基础更加坚实；随着东部地区产业向中西部加速转移，加之新一轮西部大开发和关天规划的深入实施，特别是省上专门出台支持宝鸡加快建设关天经济区副中心城市的意见，为我们加快发展提供了难得机遇。只要我们坚定信心，抢抓机遇，以“弯道超车”的勇气和魄力、“跳起来摘桃子”的精神和胆略，敢于打破常规，善于谋势赶超，就一定能实现宝鸡的科学发展、率先发展、跨越发展。

根据市第十一次党代会精神，今后五年政府工作的总体要求是：坚持以科学发展观为统领，全面贯彻党的十七届五中、六中全会精神和省、市党代会战略部署，围绕打造关天副中心、建设和谐新宝鸡、实现“六个走在前列”这一目标，以加快发展、率先跨越为总基调，全面实施“十二五”规划，坚定不移地抓好“十件大事”、加速“四个转型”，扎实推进新型工业化、农业现代化、三产规模化和城乡一体化，加快特大城市建设，深化改革开放，切实保障和改善民生，努力把宝鸡建设成为城乡经济繁荣、人民安居乐业的和谐幸福城市。

今后五年，全市经济社会发展的奋斗目标是：

——以发展速度领先、综合位次前移为目标，经济竞争力走在西部前列。到2016年，地区生产总值占到全省的八分之一，年均增速高于全省两个百分点；财税总收入达到250亿元，其中地方财政收入突破100亿元；非公经济占比达到60%，发展活力显著增强，综合实力大幅提升，基本建成关天经济区副中心城市。

——以建设国家创新型城市为核心，科技创新力走在西部前列。到2016年，全市高新技术产业产值占工业总产值的45%以上，全社会研发投入占生产总值比重达到3%以上，每百万人口发明专利授权量达到30件以上，建成国家创新型城市。

——以构建关中西部城镇群为支撑，区域辐射力走在西部前列。到2016年，城镇化率达到60%,县域经济占比达到50%以上，城市建成区面积超过110平方公里，人口达到115万以上，成为西部重要的交通枢纽，初步形成关中西部城镇群和西部商贸物流中心。

——以创建全国文明城市为统揽，文化影响力走在西部前列。到2016年，文化产业增加值占生产总值比重力争达到5%，实现文化与文物旅游深度融合、文化产业与文化事业共同繁荣，形成炎帝故里、周秦文化、东方佛都、秦岭中央公园等一批知名文化旅游品牌，成为彰显华夏文明的历史文化基地，跻身全国文明城市行列。

——以投资人居“两相宜”为重点，环境吸引力走在西部前列。到2016年，森林覆盖率达到55%，单位生产总值能耗下降16%，主要污染物排放得到有效控制，环境质量明显提高，率先在西部建成国家生态园林城市和生态市。

——以居民收入倍增和社会管理创新为突破，社会保障力走在西部前列。到2016年，全市总人口控制在390万以内，城乡居民人均收入实现翻番，社会保障体系更加完善。城乡居民的就业、教育、文化、医疗、住房等条件明显改善，社会事业主要指标达到或超过全国同期平均水平，公众安全感继续位居西部前列。

根据上述总体要求和奋斗目标，今后五年重点抓好八个方面的工作：

一是突出项目建设。坚持把项目建设作为经济工作的第一要务、重中之重，大力实施项目带动战略，用项目支撑发展规划，用项目落实思路目标，靠项目增强发展后劲。跑部进省向上争，力争更多的中省投资项目落户宝鸡；千方百计从外引，吸引一批知名企业投资兴业；发挥优势向内挖，鼓励存量资本和民间资本扩张发展。实施万亿投资计划，以大项目、大投入谋求大发展、大跨越。

二是强化招商引资。牢固树立抓招商就是抓发展的理念，把招商引资作为“一号工程”、“一把手工程”，围绕资源禀赋、产业基础、区位优势、市场潜力等，增强招商引资的针对性，大力引进国家鼓励性产业项目。到2016年，引进3-5户国内百强或世界500强企业，招商引资到位资金年均增长20%以上。

三是做大做强工业。以提高自主创新能力和市场竞争力为突破口，推进工业转型升级。持续做强大企业大集团，做大优势产业集群规模，加快工业园区发展，培育战略性新兴产业，壮大工业经济实力。到2016年，形成千亿元、五百亿元产业集群各两个，发展百亿企业集团10户，初步建成国家新材料基地、全国先进制造业重要基地、全省高端装备制造业基地。

四是加快城乡统筹。围绕构建“一主、三副、县城、重点镇、一般镇”五级城镇体系，在主城区以蟠龙塬新区、南客站片区、陈仓物流园为重点，实现新区扩张，建设新东岭等10个城市综合体，提升老城区功能品位，形成关天副中心城市基础框架；以蔡家坡、凤翔、陇县三个副中心城市为平台，加强与主城区的交通联系，完善基础设施，强化公共服务，提升载体功能，带动县城、重点镇、一般镇发展，加快城乡一体化步伐。全面落实强农、惠农、富农政策，加强农村基础设施建设，大力发展现代农业，推动农业科技进步，健全社会化服务体系，促进农业持续发展、农民大幅增收、农村和谐稳定。

五是着力改善民生。强化政策扶持，鼓励全民创业，积极扩大就业，不断增加城乡居民收入。继续完善住房、养老、医疗、残疾人等社会保障制度，健全服务网络，提升社保服务水平。实施“教育振兴八大工程”，支持有条件的县区率先实行高中阶段免费教育，三分之二县区达到省级教育强县标准。完善医疗卫生服务体系，加强食品药品监管，实现计划生育国家级优质服务先进县全覆盖，提高城乡居民健康水平。发展公益性文化事业，建成国家公共文化服务体系示范区。实施区域连片扶贫开发，改善贫困地区基础设施条件。

六是加强生态建设。持续推进绿色宝鸡创建活动，实施渭河综合治理，打造生态景观长廊，创造更加优美的人居环境。大力开展环境综合整治，改善市容市貌，提升城市品位。加大对重点生态功能区的支持力度，强力推进节能减排，发展低碳经济和循环经济，增强可持续发展能力，保护宝鸡的蓝天白云、青山绿水。到2016年，国家级生态示范县区实现全覆盖，在西部率先建成国家生态市，把宝鸡打造成宜居宜业宜商的魅力城市。

七是创新社会管理。积极探索社会管理创新的新路径、新模式，强化基层政权和城乡社区建设，夯实加强和创新社会管理的基础；健全社会治安防控、公共应急管理等机制，完善公共安全体系；加强流动人口和特殊人群管理，不断提高服务水平；正确引导网络舆论，增强虚拟社会管理能力。实施“六五”普法规划，深化“平安宝鸡”和“最安全城市”创建活动，全市社会治安满意率保持在全省前列，跻身国家社会管理创新试点城市行列。

八是深化改革开放。创新投融资机制，引进创投基金，扩大信贷规模，支持企业上市，鼓励有条件的企业发行债券，大力推进直接融资。加快国有企业改革，建立现代企业制度，不断完善国有资产监管体系。深化各类园区、管委会体制改革，构建有利于加快发展的体制机制，到2016年，高新区创建成国家级创新型科技园区。推进重点领域和关键环节改革，力争取得突破，进一步激发加快发展的活力。

2012年的主要工作

今年，是我市加快发展、跨越发展、超常规发展的重要一年，也是新一届政府谋划新发展、体现新作为、展示新形象的关键一年。全市经济社会发展的主要预期目标是：地区生产总值增长14%以上，地方财政收入增长17%，城镇登记失业率控制在4%以内，城镇居民人均可支配收入和农村居民人均纯收入分别增长14%、15%以上，全社会固定资产投资增长30%，万元GDP能耗下降 3.5%，主要污染物排放指标控制在规定范围以内，居民消费价格总水平涨幅控制在4%左右。具体做好八个方面的工作：

（一）狠抓项目建设，促进投资增长

强力推进招商引资。针对项目少、项目小的问题，制定完善招商引资优惠政策，增强投资吸引力。建立科学的工作机制，明确县区、部门引项目、争资金的任务，实行月检查、季通报制度，严格奖惩，不断提高招商引资实效。发挥工业园区、企业和驻外联络处招商引资的作用，瞄准国际国内发达地区，有针对性地组织点对点招商，实现以园引园、以企引企，在高新区、蔡家坡经开区和各县区工业园引进一批带动力强、科技含量高、税收贡献大的产业项目。年内完成合同引资700亿元，到位资金480亿元。

加快重点项目建设。针对项目进展慢的问题，提高项目审批效率，完善基础设施配套，拓宽投融资渠道，落实“五个一”工作机制，严格目标考核，千方百计加快项目进度。今年，安排市级重点项目100个，总投资1400亿元，年度投资400亿元；其中产业项目62个，年度投资196.9亿元。开工建设连霍高速市区过境段、宝汉高速西线、宝兰客专等重大交通项目，加快推进中铁宝桥扩能改造、宝钛工业园钛材加工、宝成航空精密制造工业园、农夫山泉饮用水生产线等产业项目，全年完成全社会固定资产投资1310亿元。

谋划储备一批重大项目。针对发展后劲不足的问题，结合国家投资方向和省政府53号文件，重点围绕装备制造、有色金属、农产品加工等特色优势产业，精心谋划储备一批具有较强带动力的重大项目。加大项目前期工作力度，做好宝鸡机场、合凤高速和西安—法门寺城际轨道等重大交通项目的前期工作，将发展蓝图变成项目施工图，用大项目推动大发展。

（二）发挥工业优势，增强产业支撑

提高科技创新能力。以建设创新型城市为目标，加快企业自主创新步伐，重点抓好国家石油天然气管材、油气钻井装备工程技术研究中心、高效高精度齿轮机床技术创新平台建设，争取国家数控磨床工程技术研究中心等项目，年内新增省级以上企业技术中心和工程技术研究中心5个。加大研发投入，力争规模以上企业研发投入占销售收入的比重达到2%以上。实施深水钻机与钻柱自动化关键技术研究等28个重大技术攻关和新产品开发项目，创建国家科技成果转化服务示范基地。依托重点产业和重大项目，发挥好省、市学术带头人的作用，引进培养拔尖领军人才、创新型科研团队和优秀企业家队伍。组建国家级钛及钛合金产业技术创新战略联盟，发展壮大钛材料、核级锆材等新兴产业，加速打造“宝鸡•中国钛谷”。

强化大企业大集团引领。坚持实施大企业大集团战略，落实“七优先、四激励”优惠政策和市级领导包抓机制，不断壮大骨干企业规模和实力。加快宝二电二期扩建，支持宝烟调整产品结构，完成宝石机械搬迁技改、西凤酒扩建，促进企业做大做强，年内力争东岭集团总收入突破400亿、陕汽宝鸡部分过百亿，使产值过百亿企业达到4户以上。鼓励宝钛集团、宝石钢管、宝石机械、秦川集团等龙头企业与国内外大集团开展合作，推进战略重组和行业整合，发展总部经济。积极培育一批10亿、20亿、30亿的企业，形成梯次跃进的大企业团队。加大麟北煤田开发力度，力促郭家河煤矿年产500万吨矿井达产达效，加快150万吨甲醇、麟北煤田铁路专线等项目建设进度，尽快形成新的经济增长点。

培育壮大产业集群。整合支持工业发展的各项政策和资金，大力扶持龙头企业、产业链配套企业发展。围绕九大产业集群，落实龙头企业市域内采购奖励和配套企业贷款贴息双向扶持政策，启动产业集群信息服务平台建设，促使中小企业进入龙头企业的供应网络。实施“百户配套企业成长计划”，一个龙头企业扶持5-10户主要配套企业，带动100户以上的配套企业快速发展。推进中小企业成长和全民创业工程，搭建中小企业公共服务平台，扶持中小微企业和非公经济发展，年内新增规模以上企业50户。加速工业与信息化融合、军民融合，促进民用航空、专用设备制造和新材料等军民融合产业发展。大力实施商标品牌战略，加大政策扶持和激励力度，提升西凤酒、好猫烟等品牌的知名度和美誉度。

突破工业园区发展。坚持扩大规模与加快开发并举，基础设施建设与产业开发并重，推进“两区多园”加速发展。实施高新区年产1500吨钛管生产线、太阳能光伏、西电中低压配电基地等项目，加快蔡家坡经开区金鼎15万吨铸造、通力重型车身生产、汉德车桥铸造工艺改造、华亨散热器生产线改造等15个产业项目建设，确保建成项目达产达效，促进汽车与零部件产业发展，把高新区和蔡家坡经开区打造为国家新型工业化产业示范基地。优先完善县区工业园区水、电、气、路等基础设施，突出产业特色，坚持错位发展，形成聚集互补优势，不断提升园区发展能力。

大力发展建筑业。出台扶持建筑业发展的政策措施，做大做强省二建、省七建、铁五处和市一建、市二建等龙头企业。支持四级以下建筑企业晋级提等，放手发展民营建筑企业，开拓外埠市场。支持引导外来建筑企业、施工安装企业在宝注册登记，为宝鸡发展做出贡献。强化政府监管，充分发挥建筑业协会的作用，引导行业规范健康运行。

（三）推进农业现代化，促进城乡统筹发展

改善农村基础设施。加大渭河综合治理力度，完成堤防建设任务55公里。发展节水灌溉10万亩，治理水土流失300平方公里，解决20万农村人口的饮水安全问题。加快普化水库、银洞峡水库、段家峡水库渭北灌区等项目前期工作，实施千河、嘉陵江流域治理工程，完成石头河引水一期、20座小型病险水库除险加固和5条中小河流治理任务。加快实施凤翔长青、麟游招贤等4个110千伏输变电项目，完成关中西部电网解环工程及郭家河、周塬、蔡家坡110千伏输变电项目。加快推进村镇气化工程，改善农村能源结构。切实保护耕地，大力实施中低产田改造、土地复垦整理和高标准农田建设，改善农业生产条件。完成300公里县乡油路和通村水泥路新建改建任务，提升城乡公路通行能力。

完善现代农业体系。按照“强畜、优果、稳粮”的思路，强化农业科技创新能力，加快标准化养殖基地建设，新建千头奶牛场3个、500头奶牛小区10个、200头以上生猪良繁场10个，规范提高万头生猪示范村10个，推进畜牧业突破发展。新栽苹果14万亩、猕猴桃6万亩、核桃和花椒12万亩，发展高山绿色有机蔬菜1万亩、高效设施蔬菜2万亩，做大优质果品、蔬菜基地。实施粮食增产工程，推进6个国家级、省级产粮大县建设。加大国有粮食仓储建设力度，实施农户科学储粮工程。培育壮大农业产业化龙头企业，使亿元以上企业达到25户。大力发展农民专业合作社和专业协会，提高农民组织化程度。规划建设国家级扶风休闲农业示范区，建设陈仓、太白等5个省级现代农业园区，高端乳品、生态苗木、现代烟草等12个市级农业科技示范园区，打造太白高山蔬菜、渭北塬区苹果、秦岭北麓猕猴桃、南山西山花椒、关山乔山核桃等知名品牌。加快市农产品质量安全监督检测中心、农副产品物流中心、国家级眉县猕猴桃交易中心等项目建设，完善“农社对接”、“农超对接”等新型农产品营销模式，推动政策性农业保险发展，强化农业服务体系。

加大扶贫开发力度。继续实施突破西山战略，带动北部乔山、西部关山、南部秦岭山区连片扶贫开发，编制实施麟游、千阳等5个县国家区域发展与扶贫攻坚片区规划，启动29个整村推进扶贫项目。扎实开展农村“三无户”安全住房工程、农村残疾老人生活补助、山区农民节能减柴试点工作。健全规范扶贫互助资金协会，做好信贷扶贫、产业扶贫和社会扶贫。实施雨露计划，提高贫困人口自我发展能力。利用世界银行贷款资金，改善贫困地区基础设施条件，建设扶贫项目村15个。年内全市实现6.9万农村贫困人口脱贫。

加强生态环境建设。深入开展国家生态园林城市创建活动，加快陕西西大门、南山北坡、渭河堤防绿化，实施连霍高速宝鸡段沿线环境综合整治，实现等级公路沿线林带全面贯通，新栽优质高效林2000万株，新建千里绿色长廊190公里，推进关中大地园林化建设。加大落后产能淘汰力度，关停、淘汰9户造纸、水泥等污染企业。持续开展渭河流域污染综合整治，对青岛啤酒等6户企业进行废水提标治理。实施城镇污水处理设施建设和提标改造，加快建设岐山、凤翔污水处理厂二期项目，长青等6个工业园区、绛帐镇等12个沿渭河重点镇污水处理设施。开展国家生态市创建活动，建成4个生态镇和2个生态村。着力建设农村垃圾处理和污水处理体系，不断改善城乡环境面貌，确保国家环保模范城市顺利通过复检。

（四）加快大城市建设，提升辐射带动能力

完善提升城市规划。结合关天副中心城市的战略定位和发展目标，进一步完善城市总体规划、综合交通规划、城市绿地系统规划、城市排水规划、蟠龙塬新区规划、南客站片区控制性详细规划，优化城市空间布局。强化城市规划管理，严格执行加强城市规划管理的若干规定，维护规划的严肃性和权威性。

加快扩张城市规模。加速推进城市“东扩南移北上”。“东扩”，加快陈仓物流园二期、西宝中线改扩建、陈仓老城区综合改造等项目建设；“南移”，实施南客站规划区综合开发建设，加快科技新城、南客站站房、站前广场、站前大道及安置配套工程建设进度；“北上”，加快蟠龙大道、上塬路、供水、供电、供气等基础设施建设。实施蔡家坡渭北东路、市民广场、汽车大道、城市供水系统扩建等10个市政设施项目，凤翔过境公路、时代广场、西宝北线改建项目，陇县南岸新城、主城区改造提升项目，加快蔡家坡、凤翔、陇县三个副中心城市建设。

增强城市服务功能。实施50个改造项目，重点抓好市场、小街巷、街头绿地、停车场建设。加快金台、渭滨老城区农贸市场改造提升，在高新区、代家湾等新城区建设一批农贸市场。加快金陵湾片区供水管网建设，完成代家湾地区供水服务中心工程。完善新福路、姜谭路及东岭片区排水管网，有效解决城市内涝问题。开工建设餐厨垃圾处理、城肥处理项目，新建一批公共卫生间、垃圾中转站等环卫设施。启动新东岭城市综合体项目，加快敦仁堡、姜城村等5个旧城改造项目，完善老城区服务功能。实施群众路、姜城、神农、南客站片区及虢镇城区集中供热工程，新增供热面积100万平方米。推进天然气三期利用工程，新增天然气用户1.5万户。加快10千伏城市配电网新建改造项目，完成西宝高铁供电工程。积极解决市区交通拥堵问题，完成公园路立交桥、峪泉路跨渭河人行桥工程，加快长青路、九华园市场、货场路地下停车场建设，开工金陵四桥、经二路综合改造等项目。强化市区交通管理，提升道路通行能力。大力发展公共交通，新购置公交车80辆，新建公交候车亭30座，新增公交线路2-3条，合理调整公交线路，提高公交分担率，解决市民出行难问题。

着力提高城市管理水平。推进“智慧城市”建设，完善城市管理体制，加快城市信息化进程，提高数字化城管系统运行效力，完成凤翔数字化城管试点县建设任务。强化城市管理综合执法，深入开展市容市貌、交通秩序、市场秩序整治，加强户外广告、渣土车、城市养犬管理，改善城市整体形象。加快建筑节能建设，逐步开展既有建筑供热分户计量改造。实施石鼓文化城、金陵河综合整治、渭河代家湾段生态治理项目，建成宝鸡花卉市场，美化城市环境。加强市区夜景靓化工作，完成“一河两岸”重点区域亮化任务。挖掘姜炎周秦等文化资源，建设一批文化设施，彰显城市文化特色。

加快县城和重点镇建设。完善县域城镇规划，形成优势明显、布局合理、各具特色的城镇群。加快县城建设，扩张城区规模，完善基础设施，提高建设标准，健全服务功能，提升县城品位，增强承载能力。强化蔡家坡、柳林、法门、汤峪4个省级重点示范镇基础设施和服务功能，实施73个建设项目，年内完成投资17.8亿元。进一步完善其它建制镇基础设施，增强公共服务能力。鼓励农民进城落户，引导农村富余劳动力向城镇集中，年内转移农民10万人。

（五）壮大第三产业，打造经济增长新引擎

建设西部区域商贸物流中心。调整优化商业网点布局，建设代家湾盛世广场、世纪荟萃广场、红星美凯龙家居广场等项目，支持宝商、冠森等本地知名商贸企业做大做强。改造提升餐饮、住宿、娱乐等传统服务业，建成投用万福七星国际酒店。实施宝鸡物流信息中心、宝鸡会展宾馆等项目，加快陈仓物流园商贸物流中心建设，打造综合型物流园区。推进西北、招贤煤炭物流园项目建设，扶持西部物流、华誉物流等企业发展，引进培育一批现代物流龙头企业。建立沟通协调对接机制，增加铁路客货运力，力促铁路运输实现较快增长。整合物流资源，加快运输站场、物流信息化建设，加强运输安全管理，推动交通运输业发展。

加快发展旅游业。整合旅游文化资源，编制完成旅游业发展战略规划。加大旅游业投入，完善基础设施，优化旅游发展环境。加快法门寺文化景区二期建设，全力创建国家5A级景区。完成《炎黄大帝》外景拍摄基地项目和茵香河生态治理一期工程，实施关山草原生态旅游开发、太白山国家级旅游产业新区、千湖国家湿地公园、古羌文化旅游产业示范区等25个重点项目，着力打造一批精品旅游景区。年内接待国内外游客2700万人次，实现旅游业综合收入175亿元。

积极发展文化产业。大力实施文化产业振兴工程，扶持周秦文化产业示范区等文化产业基地建设，打造一批富有宝鸡特色的文化精品。加大周原、北首岭、九成宫等遗址保护开发力度，进一步提升陈仓天地社火、凤翔泥塑等非物质文化遗产影响力，建成开放宝鸡民俗博物馆，促进文化产业与旅游、教育、科技等行业联动发展，提高文化产业对经济发展的贡献率。深入推进全国公共文化服务体系示范区创建工作，加快实施市广电中心项目，提高县级图书馆办馆标准，加强镇级文化站建设，推进广播电视村村通工程，繁荣公益性文化事业。

大力发展服务业。坚持金融服务实体经济的本质要求，继续引进银行、保险、投资基金等各类金融机构入驻宝鸡，积极稳妥发展小额贷款公司和融资性担保机构。加快推进西凤酒业、瑞科新材料、力兴钛业等企业上市步伐，完成红旗民爆、秦宝牧业首发上市。新增股份制银行、融资性担保机构3-5户，新增贷款规模100亿元以上，社会融资规模达到160亿元以上。围绕国家新材料基地和高端装备制造业基地建设，设立创投基金，促进产业发展。培育会展产业，办好“2012首届钛产业博览会”等特色会展活动。大力发展中介、创意、服务外包等新兴服务业和设计、仓储等生产性服务业，积极发展娱乐健身、市政服务等生活性服务业，提高服务业的社会化、专业化和现代化水平。

（六）深化改革开放，增强发展活力

不断深化国有企业改革。组建国有资产经营公司，有效解决国有企业融资难问题。加快秦岭化肥总厂武功、凤翔、扶风分厂改制，完成青岛啤酒重组宝鸡酒精厂任务，启动实施北照公司、五一纸厂和第一印染厂3户企业改革工作，完成宝鸡叉车五厂、市轻化总公司等企业改制重组，5户外贸企业改制取得实质性进展，市属国有企业改革取得新的突破。

稳步推进医药卫生和财政改革。以县级公立医院改革为重点，突出抓好人事分配制度、价格形成机制、服务体系建设、公共卫生服务均等化、基本药物制度推行等工作，继续推进医保支付制度改革。抓好公共财政改革，调整优化支出结构，加强对各类财政专项资金使用的绩效评估，规范资金用途，提高财政资金使用效益。加快税务信息化建设，坚持依法治税，强化税收征管，加大煤炭规费征收管理力度，做到应收尽收。

创新园区管理体制。进一步完善高新区、蔡家坡经开区、法门寺文化景区、陈仓物流园、文化旅游管委会管理体制，大胆尝试国家级、省级园区代管县区工业园的开发管理模式，年内力争蔡家坡经开区晋升为国家级经开区。借鉴眉县霸王河工业园开发模式，吸引东部地区企业整体开发建设、托管工业园区，打造产业转移示范区。

大力发展外向型经济。全方位扩大开放，支持优势企业走出去，加大对外交流合作，建设全国石油装备、机床工具、特色农产品出口基地，提高外向型经济发展水平。实施出口加工区和保税物流中心项目，推进宝鸡陆港新区建设，吸引出口导向型企业入驻，打造西北地区外贸企业集聚发展的高地。

（七）持续改善民生，提升公共服务水平

全面推进创业就业。以创建全国创业型城市为契机，整合政策资源，支持创业示范园区建设，鼓励全民创业。完善就业保障机制，解决好高校毕业生、城镇失业人员、被征地农民、复转军人、零就业家庭等重点群体的就业问题。重视和扶持劳动密集型企业发展，创造更多就业机会。强化劳动执法监察，健全维护劳动者合法权益的工作机制，构建和谐劳动关系。年内城镇新增就业5万人，转移农村劳动力100万人以上，让更多的城乡居民通过自身努力和政府帮扶富裕起来。

完善社会保障体系。进一步加强社会保障体系建设，完善 “大社保”宝鸡模式，逐步推进城乡制度并轨，进一步提高城乡居民养老、医疗等社会保险参保率，实现各项社会保险覆盖面高于全省平均水平；全面建成市县镇村（社区）四级社会公共服务平台，推进社会保障卡建设工程，为城乡居民提供便捷服务。建成社会救助动态监控系统，启动城乡低收入家庭信息比对试点工作，完善城乡低保动态管理机制。全面加快保障性住房建设，新开工保障房、棚户区改造房40498套，年内完成市区金陵湾等4个保障房小区、9县50个保障房项目共26000套。建立个人住房信息系统,精心做好保障性住房分配工作，确保公开、公平、公正。实施绛帐社会福利院迁建、市军休服务中心、市综合减灾科普教育基地、4所农村区域敬老院建设项目，启动3个残疾人托养和康复中心建设，继续为残疾人办好十件实事。支持慈善事业发展，充分发挥对社会保障的补充作用。

加快发展社会事业。大幅增加教育投入，大力实施学前教育三年行动计划，新建和改扩建公办幼儿园37所。继续实施义务教育提升工程，分层次推进标准化学校建设，深化教育教学改革，加强师德教育及业务培训，培养名教师、名校长。年内太白县创建成省级教育强县，凤县、千阳县力争创建为省级“双高双普”县。开工建设市教育中心。强化职业教育，培养一批适应产业发展的高技能人才，打响“宝鸡技工”品牌。加快市中心医院内科楼、市人民医院急救综合楼和市康复医院整体搬迁等项目建设，建成高新人民医院；继续实施乡镇卫生院“安心工程”，增加人均公共卫生服务补助经费，努力创建全国中医药工作先进市。强化医德教育和名医培养，提升医疗服务水平。扎实推进全国人口计生综合改革示范市、创建幸福家庭活动试点市等工作，启动人口和家庭公共服务中心项目，实施“母亲健康、优生促进、强基提质”三项工程，稳定低生育水平。全力做好省第十五届运动会筹备工作，加快建设宝鸡游泳跳水馆和射击馆，提早开展省运会参赛项目组队和备战训练，积极开展全国全民健身示范城市创建活动。

（八）创新社会管理，构建和谐社会

加强城乡社区建设。建立社区资源共享机制，鼓励各类文化、教育、体育等公用设施向居民开放。加大社区投入，加强社区基础设施和工作者队伍建设，发展志愿者组织，满足居民日益增长的个性化需求。加快扶风和陇县2个县级社区服务中心、60个农村社区服务中心、20个社区服务站项目建设，提升城乡社区标准化水平。规范物业管理，为居民营造舒心的生活环境。

完善社会组织管理。创新社会组织培育模式，采取政府购买服务、设立发展基金、税费减免优惠等政策措施，建立社会组织孵化基地，鼓励社会组织吸纳就业，拓展民生服务范围。加强社会组织自律与诚信建设，完善属地管理、契约管理、第三方监督和评估退出机制，促进社会组织健康发展。支持工会、共青团、妇联等群团组织发挥作用，提升社会管理整体合力。加强全民道德教育，强力推进全国文明城市创建工作。

健全公共安全体系。强化社会治安综合治理，健全网格化、立体化、智能化治安防控体系，提高公众安全感。认真办理群众来信来访，坚决完成“一控两降”任务，积极创建“三无”县区。切实加强基层调解，在征地拆迁、国企改革等工作中，充分尊重群众意愿，切实维护群众利益，有效化解各种社会矛盾。完善食品药品安全“五大体系”，年内创建国家级食品药品安全示范县1个、省级示范县2个，让群众吃得放心、用药安全。加强安全生产监管，深化煤矿、交通运输、消防等重点领域专项治理，健全以预防为主的安全生产长效机制。组织开展群众性应急演练，提高对突发公共事件的应急处置能力。抓好国防教育，加强人民防空、民兵预备役建设，巩固全国双拥模范城创建成果。继续做好民族宗教、外事、侨务、地震、气象、档案、地方志、妇女儿童和关心下一代工作，切实维护社会安定团结的良好局面。

加强政府自身建设

各位代表，加强政府自身建设，是加快发展的内在需要，更是率先跨越的根本要求。面对周边城市千帆竞发的发展态势，面对全市人民的殷切期盼，我们将以时不我待的紧迫感、慢进则退的危机感、不负众望的使命感，切实转变政府职能，全力打造务实、责任、服务、法治、诚信、廉洁政府，切实做到对人民负责、为人民服务、受人民监督、让人民满意。

坚持勤政高效，建设务实政府。坚持发展看项目、经济看效益、工作看实效，突出事关全局的重点工作和重要事项，全力以赴，真抓实干。强化无功便是过、不作为也是腐败的观念，深入开展“六扫除六确立”活动。各级政府及其组成人员要善谋大事，勇于担当，敢抓敢管，确保政令畅通无阻、政务高效运行。大力弘扬求真务实的工作作风，减少会议，精简文件，开短会、讲短话、办实事、求实效，营造激情干事光荣、平庸无为可耻的氛围。加强对公务员的学习教育，提高能力素质，在全市上下形成比学赶超、争创一流的生动局面。

坚持履职尽责，建设责任政府。责任是成就事业的重要途径。把对人民负责、让群众满意作为政府工作的根本标准，既对上负责也对下负责，既对局部负责也对全局负责，既对现实负责也对子孙后代负责，确保政府工作经得起实践、经得起群众、经得起历史的检验。进一步强化政府市场监管职能，对食品药品、质量安全、市场物价、金融市场等重点领域加强监管，确保公平竞争的市场秩序。加大对重点工作的考评，健全行政责任追究制度，建立重大事故事件政府责任部门检讨制度，加强效能督办，对不作为、乱作为的政府部门及工作人员，坚决予以严肃处理，切实提高政府执行力。

坚持执政为民，建设服务政府。以优化发展环境为突破口，强化公共服务职能，营造高效、诚信、公平的发展环境。牢记为人民服务的根本宗旨，把执政为民的要求融入进思想感情里、体现在政策措施上、落实到具体工作中，问需于民、问计于民、问政于民。各级领导干部要带头解放思想，转变作风，用人民赋予的权力，为人民谋福祉。深化行政审批制度改革，改进和完善市行政服务中心运行机制，简化办事程序，畅通项目建设和群众办事的“绿色通道”。办好“市长热线”、“政风行风热线”、政府网站和领导信箱，积极倾听群众呼声和社情民意，开展政风行风民主评议，加强与人民群众的沟通联系，努力提高为民服务水平。

坚持依法行政，建设法治政府。牢固树立法律至上的执政理念，切实做到依法治权、依法治事。全面推进依法行政，自觉接受人大的法律监督、工作监督和政协的民主监督，认真办理人大代表建议和政协委员提案。坚持政府常务会和公务员学法制度，加强教育培训，建设一支思想好、业务精、作风硬的行政执法队伍，切实做到严格、公正、文明、规范执法。深入推进科学民主决策，发挥市决策咨询委员会作用，建立决策风险评估、跟踪反馈机制，确保各项决策更加符合工作实际，更加符合科学发展的要求。

坚持诚实守信，建设诚信政府。从实际出发谋事干事，办不成的事情不说，脱离实际的目标不提，保证政策的连续性和稳定性。凡政府出台的政策和承诺的事情，必须不折不扣地落到实处，切实做到言必行、行必果，提高政府公信力。强化政务信息公开管理，保障人民群众知情权，以阳光政务取信于民。加强公务人员的诚信建设，充分发挥政府诚信的导向作用，规范商业诚信，推动社会诚信，建立信用评估体系和公示平台，努力做到信息资源共享，保证守信者得益、失信者受罚，在全社会形成诚信光荣、失信可耻的良好风尚。

坚持严格自律，建设廉洁政府。严格执行廉洁从政各项规定，加强教育、制度、监督并重的惩治和预防腐败体系建设，重点加强对社保资金、国有资产管理、政府采购、土地出让、工程招投标、项目审批等重点领域的审计和监察，从源头上预防腐败问题发生。加强节约型机关建设，规范公务接待活动，进一步压缩会议、办公、车辆等公用经费，从严控制行政费用和一般性支出，厉行勤俭节约，降低行政成本，把有限的资金用在经济发展和改善民生上。深入治理商业贿赂，坚决纠正损害群众利益的不正之风，严查违法违纪案件，确保政务规范、政事清明、政风廉洁。

各位代表，加快发展催人奋进，率先跨越正当其时。尽管在前进的道路上面临着诸多困难和挑战，但是只要我们坚定不移地向着既定目标迈进，咬紧发展不放松，就能攻坚克难、无往而不胜。让我们在市委的坚强领导下，紧紧依靠和团结全市人民，深入贯彻落实科学发展观，以更加昂扬的斗志、更加饱满的精神、更加务实的作风，锐意进取，扎实工作，为打造关天副中心、建设和谐新宝鸡、实现“六个走在前列”做出新贡献，以优异的成绩迎接党的十八大胜利召开！
